# Divergent Effects of Nitrogen Addition on Soil Respiration in a Semiarid Grassland

**DOI:** 10.1038/srep33541

**Published:** 2016-09-15

**Authors:** Cheng Zhu, Yiping Ma, Honghui Wu, Tao Sun, Kimberly J. La Pierre, Zewei Sun, Qiang Yu

**Affiliations:** 1 Institute of Animal Science and Technology, Jilin Agricultural University, Changchun 130118, China; 2 Institute of Applied Ecology, Chinese Academy of Sciences, Shenyang 110016, China; 3 National Hulunber Grassland Ecosystem Observation and Research Station/Institute of Agricultural Resources and Regional Planning, Chinese Academy of Agricultural Sciences, Beijing 100081, China; 4 Department of Biology and Graduate Degree Program in Ecology, Colorado State University, Fort Collins, Colorado 80523, USA; 5 Department of Integrative Biology, University of California, Berkeley, CA 94720 USA

## Abstract

Nitrogen (N) deposition has been steadily increasing for decades, with consequences for soil respiration. However, we have a limited understanding of how soil respiration responds to N availability. Here, we investigated the soil respiration responses to low and high levels of N addition (0.4 mol N m^−2^ yr^−1^ vs 1.6 mol N m^−2^ yr^−1^) over a two-year period in a semiarid *Leymus chinensis* grassland in Inner Mongolia, China. Our results show that low-level N addition increased soil respiration, plant belowground biomass and soil microbial biomass carbon (MBC), while high-level N additions decreased them. Soil respiration was positively correlated with plant belowground biomass, MBC, soil temperature and soil moisture. Together plant belowground biomass and MBC explained 99.4% of variation in mean soil respiration, with plant belowground biomass explaining 63.4% of the variation and soil MBC explaining the remaining 36%. Finally, the temperature sensitivity of soil respiration was not influenced by N additions. Overall, our results suggest that low levels of N deposition may stimulate soil respiration, but large increases in N availability may decrease soil respiration, and that these responses are driven by the dissimilar responses of both plant belowground biomass and soil MBC.

Human activities such as fossil fuel combustion, land use change, and fertilizer production have significantly increased nitrogen (N) input to the biosphere^1^, which has greatly altered ecosystem structure and functions^2^, including the terrestrial carbon (C) cycle. Soil respiration plays a crucial role in regulating climate-C feedbacks, which is recognized as the second largest C efflux between the atmosphere and terrestrial ecosystems^3^,^4^. Predictions from global modeling studies suggest that even a small change in soil respiration has the potential to impact atmosphere CO_2_ accumulation and the global C budget^5^,^6^. It is therefore important to understand the responses of soil respiration to N increases, which may greatly affect the direction and extent of the C balance response.

Soil respiration consists of two components, root respiration and microbial respiration. Root respiration derives from plant roots, mycorrhizal fungi, and other associated microorganisms (rhizosphere microorganisms) that use C that has been recently fixed by plant photosynthesis. Microbial respiration derives from decomposition of plant residues and soil organic matter. Previous studies have shown that N addition impacts soil respiration by altering plant above- and belowground biomass^7^ and their ratios^8^, water availability^9^, litter quantity and quality^10^, soil microbial biomass^11^,^12^, and temperature sensitivity^13^. Among these factors, plant belowground biomass has been recognized as the leading impact factor of root respiration^14^, while soil microbial biomass has been recognized as the leading impact factor of microbial respiration^11^,^12^. Thus, investigating plant belowground biomass and microbial biomass C (MBC) may be very helpful in explaining the underlying mechanisms of how N addition affects soil respiration. This better understanding of the underlying mechanisms driving the soil respiration response to increased N availability could help improve predictions of how soil C cycling may respond to N increases in the future and aid optimization of carbon-nitrogen-climate interaction models.

Past investigation of the responses of soil respiration to experimental N addition have been inconsistent^9^,^13^,^15^,^16^, because N addition could either increase root biomass^17^ or decrease C allocation to belowground biomass^8^, as well as alter soil microorganisms^18^,^19^. Changes in the relative contribution of root respiration to soil respiration could also be important in interpreting different responses of soil respiration to different levels of N addition^20^. In general, short-term studies have found that soil respiration was stimulated by relatively low levels of N additions (not more than 10 g N m^−2^)^21^, particularly in N-limited ecosystems, such as the semiarid grasslands of the Loess Plateau, Inner Mongolia, and the alpine steppe in northern Tibet^13^,^22^,^23^. In contrast, high levels of N addition (more than 15 g N m^−2^) have less consistent effects on soil respiration. For example, soil respiration was reduced in response to a high level of N addition in a mature tropical forest in southern China^16^, but increased in a temperate forest at Harvard Forest and a semiarid grassland in northern China^9^,^24^. Thus, the levels of N addition may be a dominant driver of different responses of soil respiration to N addition.

Here, we conducted an experiment with low and high levels of N addition in a semiarid grassland in Inner Mongolia, which is a part of the largest contiguous grassland in the world. We hypothesize that 1) low-level N additions should increase soil respiration, while high-level of N additions should decrease soil respiration and 2) the response of belowground biomass and MBC to N addition should highly related with soil respiration.

## Results

**Figure 1:**
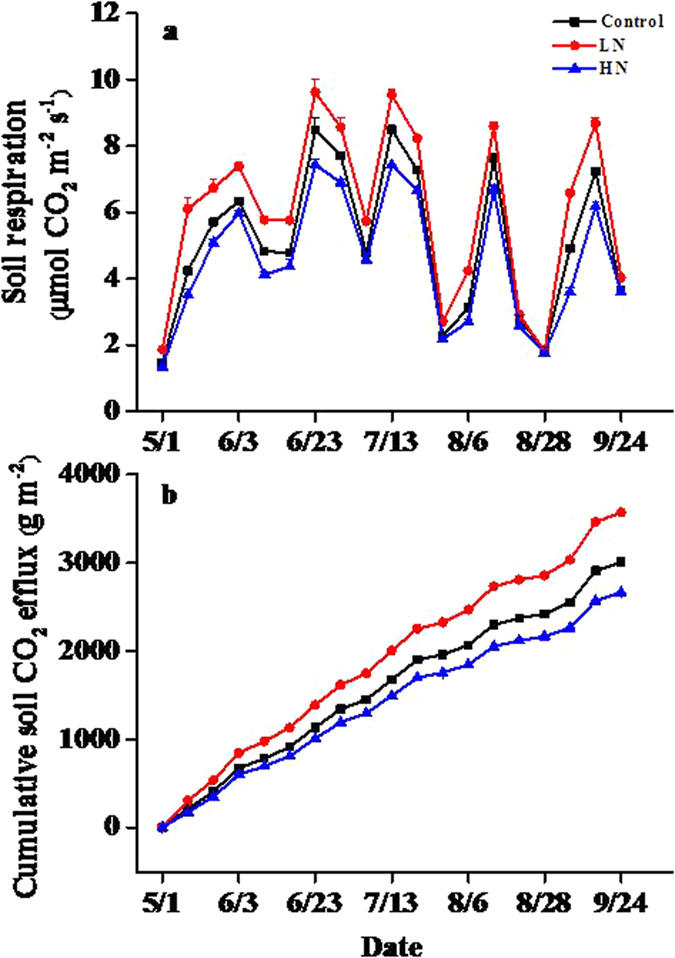
Effects of different levels of N addition on soil respiration and cumulative soil CO_2_ efflux. Control = no N added, LN = low-level N addition, HN = high-level N addition.

**Figure 2:**
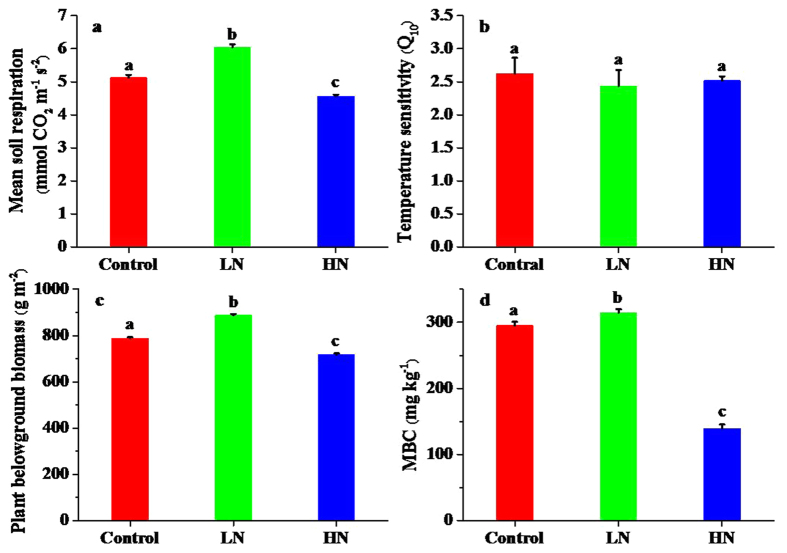
Effects of different levels of N addition on (**a**) mean soil respiration, (**b**) temperature sensitivity, (**c**) plant belowground biomass and (**d**) soil microbial biomass carbon (MBC). Control = no N added, LN = low-level N addition, HN = high-level N addition.

Soil respiration was significantly affected by N addition (*p* < 0.05) during the experimental period (Fig. 1a). The low-level N addition treatment significantly increased soil respiration (*p* < 0.05), while the high-level N addition treatment significantly decreased soil respiration (*p* < 0.05). Additionally, soil respiration showed a clear seasonal pattern, with the highest rates in late June and the lowest in early May (Fig. 1a). Nitrogen addition strongly affected cumulative soil CO_2_ efflux through its effect on soil respiration (Fig. 1b). The cumulative soil CO_2_ efflux was 18.67% higher in low-level N addition treatment plots, but 11.44% lower in high-level N addition treatment plots than in control plots. The annual mean soil respiration rate was highest under the low-level N addition treatment at 6.04 μmol CO_2_ m^−2^ s^−1^, and lowest under the high-level N addition treatment at 4.56 μmol CO_2_ m^−2^ s^−1^, with an intermediate value under the control treatment at 5.13 μmol CO_2_ m^−2^ s^−1^ (Fig. 2a). Nitrogen addition had no significant effect on the temperature sensitivity of soil respiration (*Q*_10_ values, Fig. 2b). The highest value of temperature sensitivity occurred in the high-level N addition treatment at 2.94, and the lowest value occurred in the low-level N addition treatment at 2.18.

Plant belowground biomass was significantly affected by N addition (*p* < 0.05, Fig. 2c). Mean plant belowground biomass values were 787.97 g m^−2^, 887.55 g m^−2^, and 717.51 g m^−2^ in the control, low-level N addition treatment, and high-level N addition treatment plots, respectively. Plant belowground biomass was 12.64% higher in the low-level N addition treatment and 8.94% lower in the high-level N addition treatment than in the control treatment. Similarly, soil MBC increased significantly by low-level N addition treatment, while decreased significantly by high-level N addition (*p* < 0.05, Fig. 2d). Soil MBC was 6.67% higher in the low-level N addition treatment and 55.78% lower in the high-level N addition treatment than in the control treatment.

**Figure 3:**
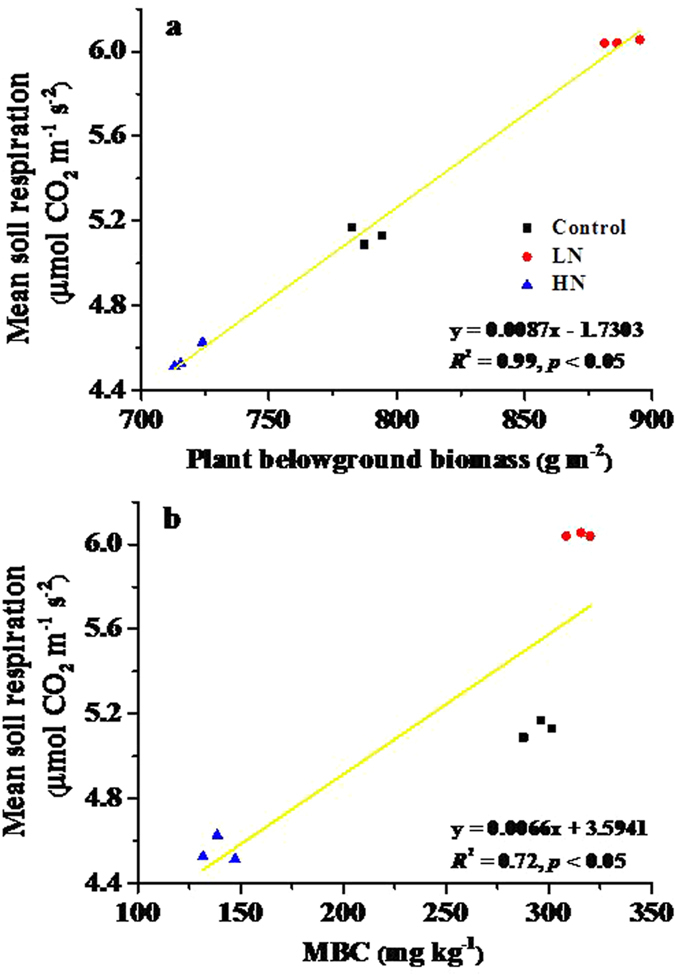
Relationship between soil respiration and (**a**) plant belowground biomass and (**b**) soil microbial biomass carbon (MBC). Control = no N added, LN = low-level N addition, HN = high-level N addition. The results of analysis showed in a and b were based on simple linear regression.

Mean soil respiration was significantly and positively correlated with both plant belowground biomass and MBC (R^2^ = 0.99, F_2,6_ = 504.9, p < 0.001; Fig. 3). Based on the relative importance analysis, plant belowground biomass explained 63.4% of the variance in mean soil respiration, while MBC explained the remaining 36.0%.

**Figure 4:**
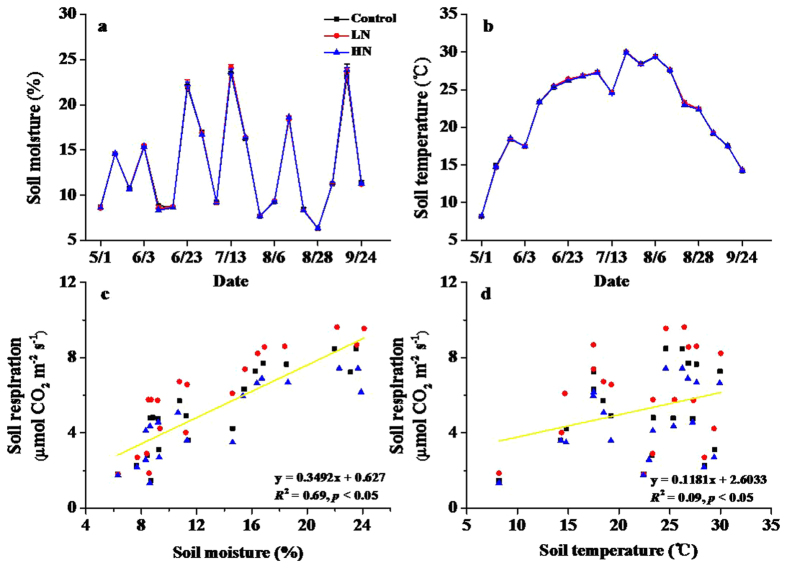
Seasonal dynamics of soil moisture, soil temperature, and their relationships with soil respiration. Control = no N added, LN = low-level N addition, HN = high-level N addition. The results of analysis showed in c and d were based on linear regression.

Both soil temperature and soil moisture showed strong seasonal variation during the experimental period (*p* < 0.05), with the highest value of soil temperature occurring in late July and the lowest value in early May (Fig. 4a,b). Mean soil temperature was 22.35 °C (range: 8.18 °C in May to 30.02 °C in July). Mean soil moisture was 13.22% (range: 6.29–24.11%) across all plots during the experiment. Nitrogen addition had no significant influence on either soil temperature or soil moisture (*p* > 0.05, Fig. 4a,b). Soil respiration was significantly and positively correlated with both soil moisture and soil temperature (R^2^ = 0.739, F_2,168_ = 238.0, p < 0.001; Fig. 4c,d). Based on the relative importance analysis, soil moisture explained 66.7% of the variance in soil respiration, while soil temperature explained the remaining 7.2%.

## Discussion

In this study, the effects of N addition on soil respiration were divergent, i.e., low-level N additions increased soil respiration and cumulative soil CO_2_ efflux, while high-level N additions decreased them. Consistent with our study, low levels of N addition have been shown to increase soil respiration in several previous studies^9^,^13^,^25^,^26^, particularly in N-limited ecosystems like our research site. In contrast, the response to N addition levels where the supply of ammonium and nitrate are in excess of the total combined plant and microbial demand (i.e., N-saturation) has been shown to be much more complex. In a mature, N-saturated tropical forest in southern China, low-level N additions showed no significant effect on soil respiration, while high-level N additions significantly reduced soil respiration^16^. Similarly, in no-till, corn-based midwestern U.S. cropping systems, soil respiration was also significantly reduced with high levels of N addition (29.1 g N m^−2^)^27^. Further, in a temperate forest, soil respiration increased after high-level N additions (15 g N m^−2^) only in the first year of treatment, then was significantly reduced with high-level N additions thereafter^24^. These responses may have occurred because high-levels of N addition can shift an ecosystem from N-limitation to N-saturation, which should negatively affect soil respiration^16^. Thus, the effects of N addition on soil respiration appear to depend on nutrient limitation and the rate of N addition.

The divergent response of soil respiration on N addition in the semiarid grassland studied here appears to mainly be mediated by plant belowground biomass and soil microbial activity. In this experiment, both microbial biomass and plant belowground biomass were highly positively correlated with soil respiration, together explaining 99.4% variation of soil respiration, indicating that they are the dominant factors that control soil respiration. Consistent with our results, significant positive correlations between soil respiration and fine root biomass as well as between soil respiration and belowground net productivity were found in cottonwood and loblolly pine plantations and a semiarid grassland, respectively^9^,^14^. These responses are likely due to the alleviation of plant and soil microbial N limitation by low-level N additions^28^,^29^, resulting in the stimulation of soil microbial respiration^30^ and plant belowground biomass.

In contrast to the positive effects of low-level N additions in our study, both microbial biomass and plant belowground biomass decreased consistently with high-level of N addition, leading to a similar decrease in soil respiration. N-induced decreases in soil respiration have previously been ascribed to decreased relative allocation of C to plant belowground biomass or reduced microbial activity and biomass with N additions, both of which are important factors affecting soil respiration^16^,^24^,^31^,^32^. In this study, plant belowground biomass contributed more to the variation of soil respiration than MBC. As plant belowground biomass contributes to root respiration and soil MBC contributes to microbial respiration, our results suggest that high-level N additions likely act to reduce both root respiration and microbial respiration, with slightly higher effects of root respiration than microbial respiration in driving the observed shift in total soil respiration. In contrast, a study in a drier grassland showed that microbial respiration responded more sensitively to N addition than root respiration^13^.

The effects of N addition on microbial and root respiration may differ with ecosystems and N conditions. Additionally, other factors such as soil pH^30^, soil dissolved organic carbon, aboveground biomass^7^, litter quantity and quality^10^, also impact the effects of N addition on soil respiration. Nitrogen saturation and decreasing soil pH are likely to occur with continuous exposure to high levels of N addition^30^,^33^, which may negatively affect plant growth due to magnesium limitation or aluminum toxicity and decreased microbial abundance, activity, and biomass, all ultimately leading to reduced soil respiration^34^,^35^,^36^,^37^,^38^. The response of soil respiration to such N additions should be closely related to the threshold point of the ecosystem^30^ beyond which ecosystem functions may shift in unpredicted ways; importantly this threshold point may differ across ecosystems^2^,^39^. Overall, plant belowground biomass and microbial biomass are dominant factors that affect soil respiration with N addition.

Soil respiration was significantly and positively related to soil temperature and soil moisture in this study, and they showed similar seasonal dynamics (Fig. 4). Consistent with our study, many previous studies have shown positive exponential or linear correlations between soil respiration and soil temperature^4^,^22^,^40^, and positive coorrelations between soil respiration and soil moisture^9^,^40^. The seasonal variation of soil respiration in our study appeared to be affected mainly by soil moisture, likely because soil moisture is the primary limiting factor for plant growth, net primary productivity, and microbial activity in the semiarid grassland of northern China^41^,^42^. However, not surprisingly, neither soil moisture nor soil temperature responded to the N addition treatments, and therefore did not affect the response of soil respiration to N additions. Therefore, soil moisture and temperature appear to be primarily important factors affecting the seasonal dynamics of soil respiration, but not the response to N availability.

In conclusion, N additions affected soil respiration divergently, with low-level N additions increasing soil respiration, while high-level N additions decreased soil respiration. Although soil temperature and soil moisture primarily explained the soil respiration dynamics, they were not affected by N additions and therefore did not drive the observed shifts in soil respiration under altered N conditions. Instead, the soil respiration response to N addition appeared to mirror changes in plant belowground biomass and MBC with the N treatments. Thus, these biotic shifts in plant belowground biomass and MBC may be the primary mechanism through which changes in soil respiration in response to N availability are manifest. Overall, low levels of N deposition may have weaker effects on C dynamics than previously believed. In contrast, high levels of N addition to natural systems, e.g., through agricultural runoff, will likely have large consequences for the C cycle and future research efforts should focus on these effects.

## Material and Methods

### Study sites and field sampling

The N addition experiment was conducted in a semiarid *Leymus chinensis* grassland (116°42′E, 43°38′N) near the Inner Mongolia Grassland Ecosystem Research Station (IMGERS) in China, which has been fenced since 1999 to prevent grazing by large animals. Mean annual precipitation at the site is approximately 350 mm, with the rainy season, during which most precipitation falls, occurring from June to August. Mean annual air temperature is −0.4 °C, and the average monthly temperature ranges from −23.0 °C in January to 17.9 °C in July from 1980–2003^30^. The vegetation is dominated by the perennial rhizomatous grass *Leymus chinensis* and the perennial bunchgrass *Stipa grandis*.

The N enrichment experiment was initiated in May 2006. Nitrogen addition treatments were established in 6 × 8 m plots, which were separated by 1 m walkways. Three replicates of each N addition treatment were established: control (without N added), low N (LN; 5.6 g N m^−2^ yr^−1^ added), and high N (HN; 22.4 g N m^−2^ yr^−1^ added), resulting in 9 experimental plots overall. Nitrogen was added in the form of urea (CO(NH_2_)_2_), which was thoroughly mixed with sand and evenly spread across the plot surface in May 2006 and 2007, following a rainfall event each year. To ensure N was only the limiting nutrient, 1.55 g P m^−2^ yr^−1^ was added to the N addition treatment plots as KH_2_PO_4_^43^.

### Soil respiration

Three PVC collars (5 cm in height and 11 cm in diameter) were inserted into the soil in each plot, with the bottom of the collars buried 3 cm below the soil surface. Soil respiration was measured approximately once per week from 1^st^ May to 23^rd^ September in 2007 with an LI-6400 portable photosynthesis system attached to a soil CO_2_ flux chamber (991 cm^3^ in total volume; LI-COR 6400-09 TC, LI-COR Inc., Lincoln, NE, USA). Soil respiration measurements were made on the third day after a rainfall event, to avoid the confounding effects of precipitation on soil respiration to the extent possible. The measurements were conducted between 8:00 am and 12:00 pm, and each measurement was completed within 2 min.

### Soil moisture and soil temperature

Discrete soil temperature and volumetric soil moisture measurements were made concurrently with soil respiration measurements. Soil temperature was measured using a thermocouple probe connected to a LI-6400. The probe was inserted into the soil to a depth of 5 cm in a location adjacent to the PVC collar. Volumetric soil moisture was determined at a range of depths from 0–10 cm using a TRIME TDR probe (IMKO, Ettlingen, Germany) in the same location where soil respiration was measured.

### Plant belowground biomass

Plant belowground biomass was estimated from 1 m deep soil cores collected on 28^th^ August 2007 with a soil auger (7 cm inner diameter). Three soil cores were taken from each plot and sufficiently mixed to create a homogenous sample. To ensure there was no clay sticking to the roots, the roots were washed three times over a 0.5-mm sieve. All biomass samples were oven-dried to a constant weight at 85 °C.

### Soil microbial biomass carbon

Microbial biomass C (MBC) was determined using the fumigation extraction technique^44^. Soil samples were collected from each plot after the plant biomass harvest. Each sample comprised five soil cores (3 cm in diameter and 10 cm in depth) and were placed in individual plastic bags and then immediately stored at 4 °C. Soil samples from single sampling cores were divided into paired subsamples of 10 g each. One subsample was immediately extracted with 20 ml 0.5 M K_2_SO_4_ for 60 min on a rotary shaker at 150 rpm. The second subsample was fumigated under chloroform vapor for 24 h in a desiccator. followed by ten vacuum/release purge cycles. and then extracted as described above. Extracts were filtered using a 0.2 lm syringe filter and analyzed for total organic carbon (TOC) using a TOC analyzer (Dimatec Analysentechnik GmbH, Essen, Germany). MBC was calculated as the difference in TOC between the two subsamples.

### Statistical analysis

Statistical significance was defined at the 95% confidence level (alpha = 0.05). Repeated measures ANOVAs were performed to examine the effects of N additions on soil respiration, soil temperature and soil moisture, and standard ANOVAs were performed to examine the effects of N additions on plant belowground biomass and MBC. Cumulative soil CO_2_ (g CO_2_ m^−2^) efflux was estimated as the sum of efflux during the days between sampling dates.

A multiple regression was used to examine the effects of soil temperature and soil moisture on soil respiration with the following linear equation:


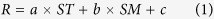


where *R* is the soil respiration, *ST* is the soil respiration, *SM* is the soil moisture and *a*, *b* and *c* are coefficients. *Q*_*10*_ values were obtained as follows:


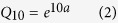


where coefficient *a* was from Eq. (1)^45^.

Two linear multiple regressions were performed to determine the mechanisms underlying the soil respiration responses to N additions. First, mean soil respiration was regressed against plant belowground biomass and MBC. Second, the time series data was used to examine the relationships between soil respiration and soil temperature and soil moisture. The strengths of the relationships within each regression were assessed by comparing the R^2^ contribution of each regressor averaged over all possible orderings of the regressors in the model using the relaimpo package in R. Regressions incorporating non-linear terms were also tested, but rejected in favor of the models described above based on AIC.

All ANOVAs were performed using SPSS 19.0 for Windows (USA) and all regressions were performed using R v. 3.2.4^46^.

## Additional Information

**How to cite this article**: Zhu, C. *et al*. Divergent Effects of Nitrogen Addition on Soil Respiration in a Semiarid Grassland. *Sci. Rep.* **6**, 33541; doi: 10.1038/srep33541 (2016).
